# CtIP Is Required to Initiate Replication-Dependent Interstrand Crosslink Repair

**DOI:** 10.1371/journal.pgen.1003050

**Published:** 2012-11-08

**Authors:** Michelle L. Duquette, Qingyuan Zhu, Ewan R. Taylor, Angela J. Tsay, Linda Z. Shi, Michael W. Berns, Clare H. McGowan

**Affiliations:** 1 Department of Molecular Biology, The Scripps Research Institute, La Jolla, California, United States of America; 2 Institute of Engineering in Medicine, University of California San Diego, La Jolla, California, United States of America; 3 Beckman Laser Institute and Department of Biomedical Engineering, University of California Irvine, Irvine, California, United States of America; 4 Department of Cell Biology, The Scripps Research Institute, La Jolla, California, United States of America; Stanford University School of Medicine, United States of America

## Abstract

DNA interstrand crosslinks (ICLs) are toxic lesions that block the progression of replication and transcription. CtIP is a conserved DNA repair protein that facilitates DNA end resection in the double-strand break (DSB) repair pathway. Here we show that CtIP plays a critical role during initiation of ICL processing in replicating human cells that is distinct from its role in DSB repair. CtIP depletion sensitizes human cells to ICL inducing agents and significantly impairs the accumulation of DNA damage response proteins RPA, ATR, FANCD2, γH2AX, and phosphorylated ATM at sites of laser generated ICLs. In contrast, the appearance of γH2AX and phosphorylated ATM at sites of laser generated double strand breaks (DSBs) is CtIP-independent. We present a model in which CtIP functions early in ICL repair in a BRCA1– and FANCM–dependent manner prior to generation of DSB repair intermediates.

## Author Summary

One of the most lethal forms of DNA damage is the interstrand crosslink (ICL). An ICL is a chemical bridge between two nucleotides on complementary strands of DNA. An unrepaired ICL is toxic because it poses an unsurpassable block to DNA replication and transcription. Certain forms of cancer treatment exploit the toxicity of ICL generating agents to target rapidly dividing cells. Sensitivity to crosslinking agents is a defining characteristic of Fanconi Anemia (FA), a hereditary syndrome characterized by an increased risk in cancer development and hematopoietic abnormalities frequently resulting in bone marrow failure. The mechanism underlying ICL repair is important to human health; however, the sequence of molecular events governing ICL repair is poorly understood. Here we describe how the repair protein CtIP functions to initiate ICL repair in replicating cells in a manner distinct from its previously described role in other forms of DNA repair.

## Introduction

Cellular DNA can be chemically modified and damaged when exposed to environmental agents, metabolic byproducts, or chemotherapeutic agents. The most toxic of these lesions is the interstrand crosslink (ICL), a covalent bridge formed between complementary strands of DNA. If not repaired, ICLs prevent DNA strand separation resulting in a block to replication and transcription. ICL generating agents are commonly used in the treatment of cancer. Sensitivity to crosslinking agents is a defining characteristic of Fanconi Anemia (FA), a rare hereditary syndrome characterized by an increased risk in cancer development and hematopoetic abnormalities frequently resulting in bone marrow failure [1]. Elucidation of the cellular pathways that repair ICLs is highly relevant to understanding carcinogenesis, development of novel therapies to treat FA patients, and to the development of better targeted chemotherapeutic drugs.

Sensitivity assays suggest that eukaryotic cells have evolved multiple complex systems to repair ICLs that involve the intersection of several different repair pathways (reviewed in [2], [3]). However, the specific mechanism by which ICLs are detected and repair is initiatedremains unknown. A major ICL repair pathway in higher eukaryotes functions during S-phase and is thought to be replication dependent [4]–[7]. ICLs can also be repaired in a replication independent manner [8]–[10]. Current models of replication mediated ICL repair, suggest that repair is initiated when a fork stalls due to encountering an ICL [6], [11]. FANCM/FAAP24 then binds to the ICL stalled fork [12]–[17]. Next, single stranded DNA (ssDNA) is generated and bound by RPA [11], [15], [18] and the DNA damage response kinase ATR/ATRIP localizes to the damaged chromatin through binding to RPA [19]. Localization of ATR/ATRIP to damaged DNA is essential for activation of the S-phase checkpoint and ICL repair [20], [21]. The ability of ICLs to activate the checkpoint is dependent on the FA core complex (FANCA/B/C/E/F/G/M) [10], but not FANCI-FAND2 [18]. The generation of ssDNA at stalled replication forks is thought to be critical for ATR activation. However, the factors required to generate ssDNA under circumstances in which the ICL poses a structural barrier to helicase uncoupling from the DNA polymerase at the replication fork are not known [22]. It has been shown ssDNA arises at an ICL stalled fork in *Xenopus* extracts due to resection of the lagging strand [11]. In addition this ssDNA is competent for Rad51 loading prior to generation of a DSB ICL repair intermediate [23].

The FANCI-FANCD2 complex is phosphorylated by activated ATR in response to ICL stalled replication forks [20], [24], [25]. This phosphorylation facilitates FANCI-FANCD2 monoubiquitination by the FA core complex [24], [25]. Monoubiquitination is essential for localization of the FANCI-FANCD2 complex to damaged chromatin where it directs downstream repair steps [18], [26]–[28]. The FANCI-FANCD2 complex is required for the initial ICL incision step in replication competent *Xenopus* extracts [18].

Several candidate nucleases have been identified that may function to excise the ICL. These nucleases include XPF/ERCC1, MUS81/EME1, their regulator SLX4 (also known as the Fanconi Anemia gene *FANCP*) [29]–[37], SNM1A [37], [38] and SNM1B [39]. Whether they act redundantly or in concert is not clear, however they are all characterized by showing higher sensitivity to crosslinking agents compared to other forms of damage. MUS81/EME1 and XPF/ERCC1 have been proposed to function both early [40], [41]and late in the ICL repair process [29]–[32], [42], [43]. The nuclease FAN1 also confers resistance to ICL inducing agents and has been shown to interact physically with the monoubiquitinated form of the FANCD2 nuclease [44]–[46]. Although a large number of nucleases are implicated in ICL repair, it remains unclear at which specific step these proteins exert their functions.

Following ICL incision, translesion synthesis by an error prone polymerase repairs the strand opposite the incised ICL [11], [47]–[50]. Excision of the ICL and polymerase extension up to the excised region results in the generation of double stranded ends resembling a double strand break (DSB). The DSB is repaired by homologous recombination as evidenced by the extreme sensitivity of homologous recombination impaired cell lines to ICL inducing agents [51]–[53].

We hypothesized that CtIP might play an important and unique role in replication dependent ICL repair for the following reasons. First, studies of the CtIP homologs in *A. thaliana (AtGR1)* and *S. cerevisiae* (*SAE2*) have revealed a conserved role for CtIP in ICL repair due to their ability to confer resistance to the ICL inducing agent mitomycin C (MMC) [54], [55]. Second, CtIP expression is enhanced in S through G2 phase [56], [57] when ICL repair primarily occurs. Third, CtIP plays a conserved role in coordinating the recognition and resection of double strand breaks with the MRE11-RAD50-NBS1 (MRN) complex [58]–[63] and may act to facilitate the generation of ssDNA ends following ICL incision.

To investigate the mechanism of ICL repair in live mammalian cells we developed a system that takes advantage of the chemical and physical characteristics of the cross-linking agent, 8-methoxy psoralen (8-MOP). 8-MOP intercalates into DNA and forms DNA ICLs upon exposure to long wavelength UVA light (365 nm) [64]. In this system we used laser directed 2-photon activation of 8-MOP via infrared light (730 nm) to generate ICLs in individual human S-phase nuclei. This approach restricts damage to precisely defined subnuclear volumes allowing one to monitor the spatiotemporal dynamics of DNA repair factors after ICL induction. This system was used to determine what role CtIP plays in ICL repair. We show that CtIP plays an important and unexpectedly early role in ICL repair in S phase human cells. In addition to the known role of CtIP and MRE11 in DSB end resection [59], [60], [62], the data presented here demonstrates that CtIP plays an additional role in ICL repair prior to generation of DSB repair intermediates. The data show that CtIP acts prior to RPA loading and localization of ATR and FANCD2 to ICL containing DNA. CtIP is required for the accumulation of known DSB repair factors phospho-histone H2AX (γH2AX), and ATM autophosphorylated on serine-1981 (ATM-pS1981) at ICLs, but not at DSBs. The data support a model in which CtIP is recruited to ICLs in a FANCM dependent manner and is required to initiate ICL processing of stalled forks prior to ICL incision.

## Results

### CtIP depletion sensitizes cells to ICL inducing agents

**Figure 1:**
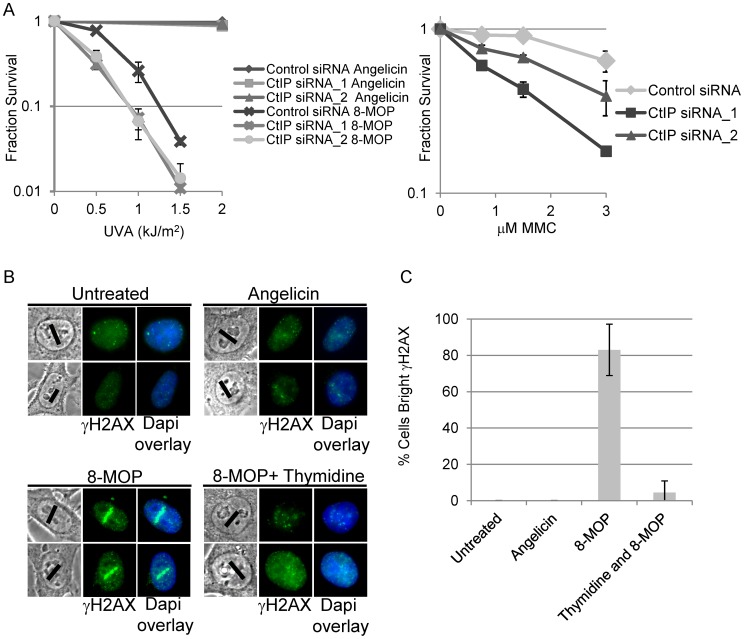
CtIP depletion sensitizes cells to ICL inducing agents. (A) Left, Survival of HEK293 cells transfected with control or CtIP siRNAs and exposed to 8-MOP+UVA or angelicin+UVA. CtIP_1 and CtIP_2 are two independent siRNAs. Cells were treated with indicated drug 48 hours post transfection and survival was assessed 8 days post irradiation. Fraction surviving cells is calculated in respect to untreated cells. Right, Survival of control or CtIP depleted cells treated 8 days following exposure to indicated concentration of MMC. Bars indicate standard deviation between 3 independent experiments. (B) γH2AX staining of fixed HeLa cells 2 hours post microirradiation with 730 nm laser light. S-phase synchronized cells were treated with indicated drugs prior to laser microirradiation. Black line on phase images indicates region of nucleus targeted by the laser. Thymidine arrested cells were microirradiated in the presence of 8-MOP. (C) Quantification of cells scored as having bright γH2AX staining along laser tracks. For details on cell analysis see Experimental Procedures. Bars indicate standard deviation between 3 independent experiments.

To determine if CtIP plays a role in ICL repair, we assayed CtIP depleted human cells for sensitivity to the ICL inducing agents MMC or 8-MOP plus whole cell UVA irradiation (365 nm). The products of activated 8-MOP on DNA are predominantly ICLs, but monoadducts are also formed [64], [65]. The psoralen angelicin, which is structurally related to 8-MOPs, forms monoadducts upon UVA exposure and was used to control for cellular sensitivity to DNA monoadducts [66]. CtIP was depleted from Human Embryonic Kidney Cells (HEK293) by transient transfection of two previously described independent small interfering RNAs (siRNAs) [59]. Luciferase siRNA was used as a nontargeting control. CtIP RNA levels were monitored by RT-qPCR and were found to be reduced on average by 70%. Sensitivity to ICL and monoadduct inducing compounds was determined by assessing the fraction of surviving cells after 8 days. As shown in Figure 1A, CtIP depleted cells were sensitive to the ICL inducing agents 8-MOP+UVA and to MMC. CtIP depleted cells were not sensitive to angelicin plus UVA. CtIP depleted cells were only slightly sensitive to IR induced DSBs (Figure S1). This sensitivity profile is similar to that reported for *A. thaliana AtGR1/AtCom1* mutated cells [55], and suggests that CtIP plays an important and conserved role in ICL repair.

### Generation of ICLs by 2-photon activation of 8-MOP

To define the role of CtIP in ICL repair, we established a system that enables the examination of the spatial and temporal recruitment and retention of repair proteins at region specific ICLs. An analogous approach has been used to generate study ICL repair using a UV laser plus light activated psoralen [9], [67]. Our approach uses a near infrared femtosecond laser to produce 730 nm light which activates 8-methoxypsoralen (8-MOP) by two-photon activation (effective wavelength of 365 nm at the focal point). This enabled us to define precisely a subnuclear volume in which DNA damage was created while avoiding damage outside the focal points [68]. This method allowed specific activation of 8-MOP with doses of light that caused no detectable damage in psoralen free control cells (Figure 1B).

To define the optimal laser power, we first identified the lowest laser power that gave a robust γH2AX signal in cells treated with 8-MOP, and no observable γH2AX by laser alone (Figure 1B). Cells were subject to thymidine block and release to enrich for replicating cells [69]. One hour after thymidine release the indicated drug was added, and cells were microirradiated with 730 nm light using a femtosecond laser. Following microirradiation, cells were maintained in culture for 2 hours allowing time for replication forks to encounter the damage prior to fixation (Figure S2). Angelicin was used as a control for the amount of γH2AX at the laser tracks that resulted from the generation and/or processing of monoadducts. To verify that the γH2AX along laser tracks was replication dependent a control experiment was performed in which cells were held in thymidine during drug treatment and laser microirradiation. γH2AX staining signal along microirradiated tracks was quantified in individual cells using Image J (See Materials and Methods). The proportion of cells with positive γH2AX staining along microirradiated tracks are summarized in Figure 1C. The number of cells scored positive for γH2AX signal along laser tracks was highly increased in S-phase cells microirradiated in the presence of 8-MOP (83%) relative to microirradiated drug free cells (0%) (n = 29 and 25 cells respectively). Blocking replication in 8-MOP microirradiated cells reduced the number of γH2AX positive cells almost to background levels (4.5%) (n = 21). Angelicin treated microirradiated cells did not yield bright γH2AX signal (0%) (n = 25) (Figure 1B, 1C), however we do not exclude the possibility that adduct formation by angelicin may be less efficient than that of 8-MOP. Thus we conclude that the γH2AX signal detected in 8-MOP microirradiated cells is primarily due to the replication dependent processing of ICLs.

**Figure 2:**
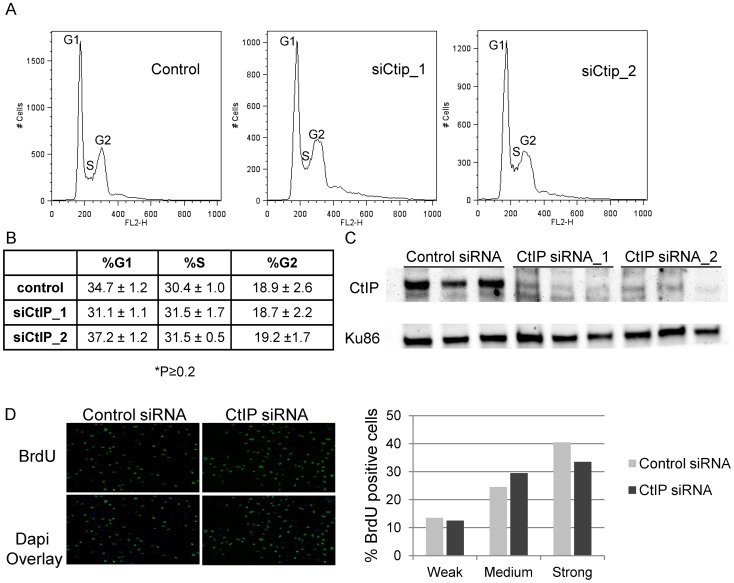
Effects of depletion of CtIP on cell cycle and BrdU incorporation. (A) Cell cycle profiles of control and siCtIP transfected HeLa cells 48 hours after transfection. Labels mark the cell populations in the G1 (left peak), S (saddle), and G2 (right peak) phases of the cell cycle based on their DNA content. (B) Summary of cell cycle distributions in control and siRNA transfected HeLa cells. Percentages shown are averages of the results of 3 experiments ± the standard deviations. There was no significant difference between the cell cycle distributions of control and CtIP depleted cells (P≥0.2). (C) Immunoblot analysis confirming knockdown of CtIP by two independent siRNAs in 3 independent experiments. Protein extracts were prepared at 48 hours post transfection. Blot was probed with anti-CtIP antibody as indicated on left. A Ku86 was used as a loading control. (D) BrdU incorporation is unaffected in CtIP depleted cells. siRNA transfected HeLa cells were synchronized with thymidine, were released into S-phase 48 hours post transfection, and fixed after 20 minutes BrdU incorporation. Cells were stained for BrdU. Left, Images of fixed control and CtIP depleted cells following staining for BrdU. Right, Quantification of relative BrdU signal in control and CtIP depleted cells.

Previously published reports have shown that CtIP plays a role in G1/S progression in mouse fibroblasts (MEFs, NIH 3T3) [57], [70]. We wanted to examine whether CtIP depletion affected cell cycle and S-phase progression in HeLa which could have an effect on the efficiency of ICL repair. Cell cycle analysis was performed on CtIP depleted and control HeLa cells. No significant difference in cell cycle distribution was observed between control cells and 2 independent CtIP siRNAs in 3 independent experiments (Figure 2A, 2B). Figure 2C shows that both CtIP siRNAs effectively depleted CtIP in the samples analyzed for cell cycle distribution. Quantification of the nucleotide analog bromodeoxyuridine (BrdU) incorporation in individual siRNA transfected cells was determined to compare the number of S phase cells and the relative rates of replication associated nucleotide incorporation under the same conditions used in laser experiments. ICL detection and initiation of repair in S phase occurs primarily when a replication fork encounters and stalls at an ICL. Therefore it was important to verify that fork progression, as determined by BrdU incorporation, is not affected in CtIP depleted cells relative to control cells. As judged by quantification of pixel intensities in individual cells there was no significant difference in replication rate between control and CtIP depleted cells Figure 2D. In addition, under the conditions used for laser irradiation experiments >95% of the cells were in S phase. The lack of an effect on cell cycle distribution or BrdU incorporation in CtIP depleted HeLa cells suggests that the decrease in γH2AX generation at ICLs in CtIP depleted cells is due to a defect in ICL processing as opposed to a cell cycle progression defect.

### CtIP is required for histone H2AX phosphorylation at laser-activated ICLs

To determine when CtIP acts in the ICL repair process, known markers of the DNA damage response and repair process were monitored in CtIP depleted HeLa cells. The first marker examined was γH2AX. To compare the function of CtIP in ICL repair to its known role at directly generated DSBs [59], [62], parallel experiments were conducted in which DSBS were generated directly by microirradiation with 532 nm laser light (64). Laser doses for 730 nm and 532 nm light were predetermined to use the minimal doses that give readily detectable γH2AX signals 2 hour post microirradiation. (Figure S2).

**Figure 3:**
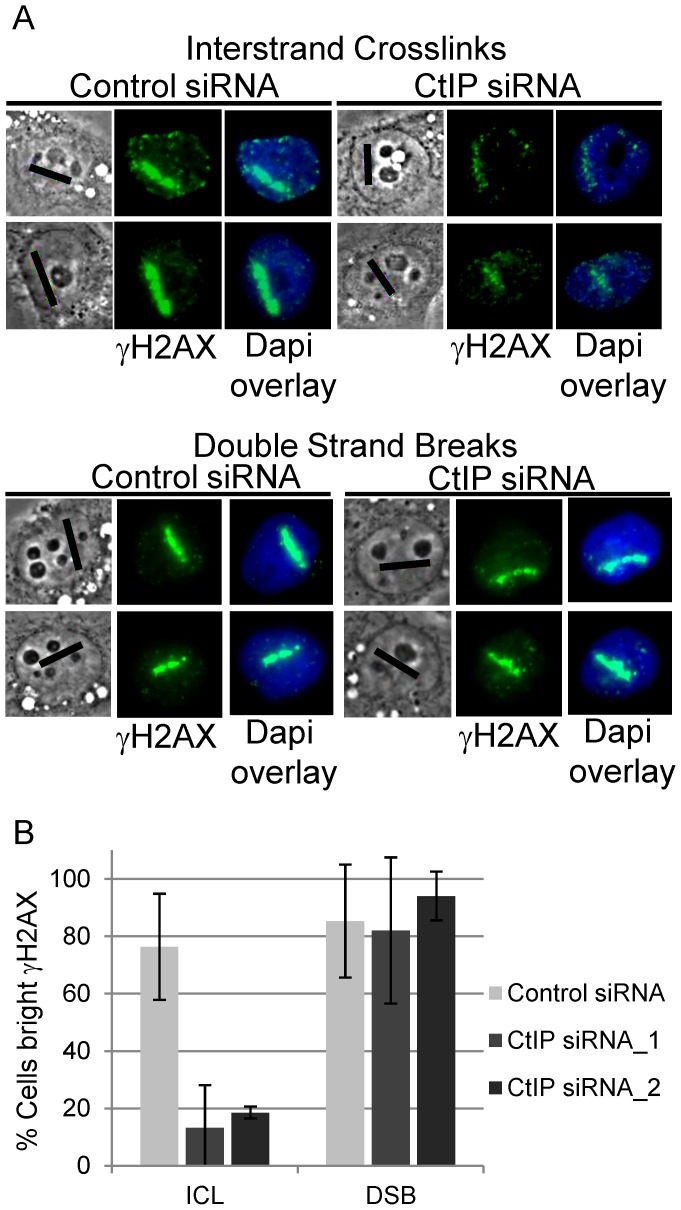
CtIP depletion reduces γH2AX at ICLs but not at DSBs. (A) γH2AX staining of S-phase control and CtIP depleted cells 2 hours post microirradiation with 730 nm laser light in the presence of 8-MOP to form ICLs (above) or 532 nm laser light to form DSBs (below). (B) Quantification of cells scored as having bright γH2AX staining along laser tracks in control (light grey), CtIP siRNA_1(grey) and CtIP siRNA_2 (dark grey) depleted cells. Bars indicate standard deviation between 5 independent experiments.

γH2AX phosphorylation was found to be strongly reduced in CtIP depleted S-phase HeLa cells along ICL containing microirradiated tracks (Figure 3A, top panel). In contrast, CtIP depletion had no detectable effect on H2AX phosphorylation at DSBs induced directly by laser irradiation (Figure 3A, bottom panel). The percentage of cells containing bright γH2AX signal, as determined by quantification of pixel intensity in individual cells along ICL containing laser tracks, was reduced significantly, over 4 fold, in cells depleted with 2 independent siRNAS targeting CtIP (13% and 19% respectively) relative to control cells (76%), (n = 48, 29 and 66 cells respectively; P-values = 0.003 (CtIP siRNA_1) and 0.009 (CtIP siRNA_2) (Figure 3B). In contrast, the percent cells with bright γH2AX along DSB containing laser tracks was not reduced in CtIP depleted cells (CtIP siRNA_1 82%, CtIP siRNA_2 94%) relative to control cells (85%), (n = 19, 19 and 26 cells respectively; P-value > = 0.5). The differential effects of CtIP depletion on H2AX phosphorylation at ICLs and DSBs suggests that CtIP plays a role in ICL repair that can be distinguished from its role in DSB repair.

 To examine the function of CtIP relative to MUS81 and XPF, two nucleases that have been suggested to act early in ICL repair [40], [71], the effects of MUS81 and XPF depletion on γH2AX accumulation at ICLs was examined. Depletion of either XPF or MUS81 did not have a measurable effect on γH2AX accumulation along ICL containing laser tracks (Figure S3). This indicates that Mus81 and XPF function downstream of γH2AX generation in ICL repair.

### CtIP is required for ATM phosphorylation at ICLs

ATM becomes activated in an MRN dependent manner and phosphorylates H2AX at DSBs early in DSB repair [72]–[74], while ATR contributes to ATM activation and H2AX phosphorylation as a result of replication stress [75]–[77]. ATM has also been found to function in the FA pathway [24]. To test if CtIP is required for ATM phosphorylation at ICLs, and thus functions upstream of DSB generation, we examined the effects of CtIP depletion on phosphorylation at serine-1981, a characterized autophosphorylation site. [72].

**Figure 4:**
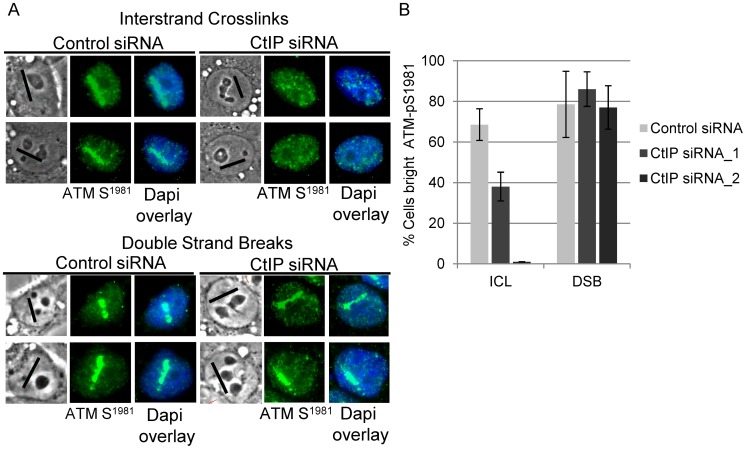
CtIP depletion reduces ATM-pS1981 at ICLs but not at DSBs. (A) ATM-pS1981 staining of S-phase control and CtIP depleted cells 2 hours post microirradiation with 730 nm laser light in the presence of 8-MOP to form ICLs (top) or 532 nm laser light to form DSBs (below). (B) Quantification of cells scored as having bright ATM-pS1981 staining in control (light grey) CtIP siRNA_1(grey) and CtIP siRNA_2 (dark grey) depleted cells. Bars indicate standard deviation between 3 independent experiments.

Consistent with the premise that DSBs are formed as an intermediate during ICL repair, ATM-pS1981 signal was readily detectable in control cells treated with 8-MOP and irradiated to form ICLs. In contrast, ATM-pS1981 signal along ICL containing laser tracks was reduced significantly in CtIP depleted S-phase HeLa cells (38%) relative to control cells (69%), (n = 32 and 27 cells respectively; P-value = 0.05) (Figure 4A, upper panel). By contrast, CtIP depletion did not significantly reduce the number of cells with bright ATM-pS1981 along DSBs (86%) relative to control cells (79%) (Figure 4A, lower panel), (n = 33 and 29 cells respectively; P-value > = 0.1) (Figure 4B).

### CtIP is required for FANCD2 accumulation at ICLs

**Figure 5:**
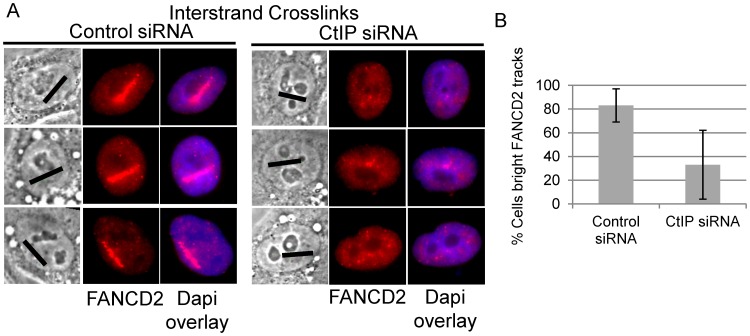
CtIP depletion reduces FANCD2 at ICLs. (A) FANCD2 staining of S-phase control and CtIP depleted cells 2 hours post microirradiation with 730 nm laser light in the presence of 8-MOP. (B) Quantification of cells scored as having bright FANCD2 staining along laser tracks in control or CtIP depleted cells.

Biochemical analysis has demonstrated that the FANCI-FANCD2 complex is required for the incision of interstrand crosslinks in replication competent *Xenopus* extracts [18]. We therefore examined whether FANCD2 was properly localized to laser activated ICLs in CtIP depleted cells. FANCD2 accumulation was found to be reduced along ICL containing microirradiated tracks in CtIP depleted cells (Figure 5A). The number of cells containing bright FANCD2 signal was reduced significantly by 2.7 fold in CtIP depleted cells (33%) relative to the control cells (88%) (n = 24 and 26 respectively; P-value = 0.026) (Figure 5B). These results demonstrate that CtIP acts upstream of FANCD2 localization to chromatin and point to a role for CtIP in ICL repair prior to ICL incision and unhooking.

### CtIP is required for RPA and ATR accumulation at ICLs

**Figure 6:**
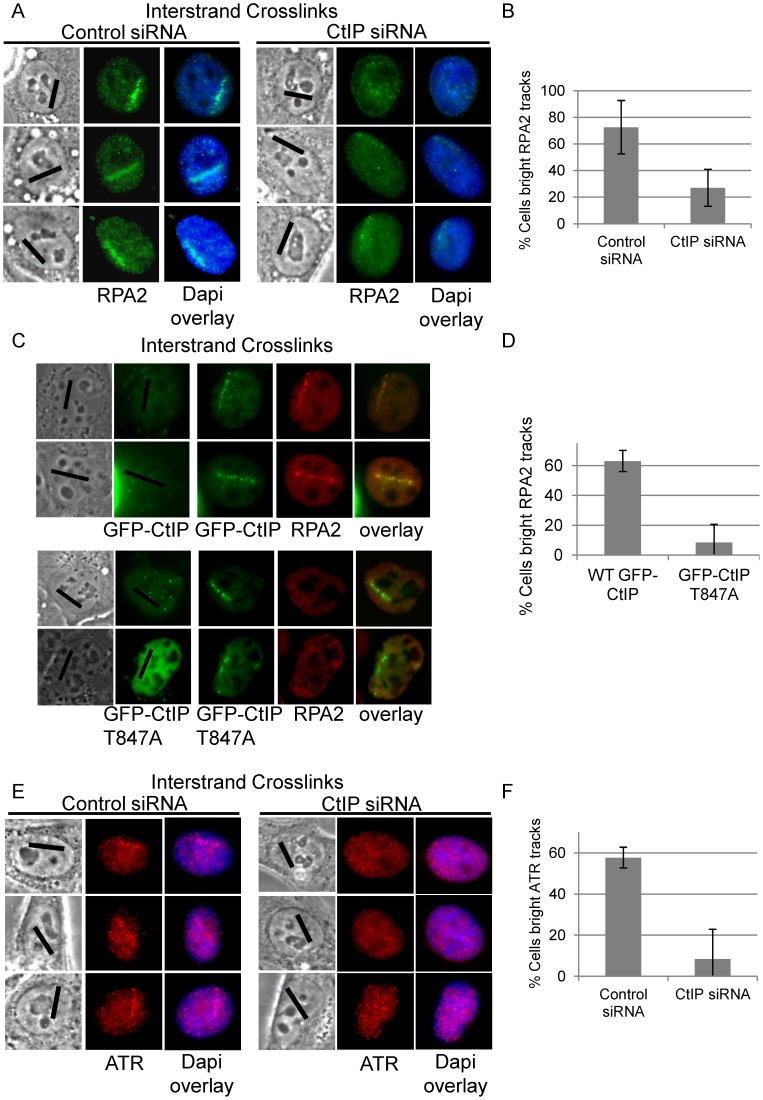
CtIP depletion reduces RPA and ATR accumulation at ICLs. (A) RPA staining of S-phase control and CtIP depleted cells 2 hours post microirradiation with 730 nm laser light in the presence of 8-MOP. (B) Quantification of cells scored as having bright RPA staining intensity along laser tracks in control or CtIP depleted cells. (C) GFP-CtIP or GFP-CtIPT847A in S-phase cells pre pre-and post-microirradiation with 730 nm light in the presence of 8-MOP. Cells were stained for RPA2. (D) Quantification of RPA2 along laser tracks in cells scored positive for GFP-CtIP or GFP-CtIPT847A accumulation. (E) ATR staining of S-phase control and CtIP depleted cells 2 hours post microirradiation with 730 nm laser light in the presence of 8-MOP. (F) Quantification of cells scored as having bright ATR staining along laser tracks in control or CtIP depleted cells. Bars indicate standard deviation between 3 independent experiments.

ATR phosphorylates the FANCI-FANCD2 complex which is required for its monoubiquitination and localization to ICLs [20]. RPA coated ssDNA is required for ATR activation [19] and CtIP/MRN is required for resection at double strand breaks [59], [62]. We hypothesized that CtIP might facilitate resection of DNA ends present at an ICL stalled replication fork. Therefore we examined RPA2 accumulation at ICL containing microirradiated tracks in CtIP depleted cells compared to control cells. RPA2 accumulation at laser tracks was found to be strongly reduced in CtIP depleted S-phase HeLa (Figure 6A). The percent cells containing bright RPA2 tracks was reduced 2.7 fold in CtIP depleted cells (27%) relative to control cells (73%) (n = 29 and 41 cells respectively, P value = 0.005) (Figure 6B).

CtIP is targeted for phosphorylation at Thr847 by cyclin dependent kinase (CDK) [63]. Phosphorylation at this site is required to promote resection at DSBs, but is not required for CtIP recruitment to DSBs [63], [78]. We tested whether a Thr-847 to Ala CtIP mutation would also affect resection at ICLs. Endogenous CtIP was depleted by siRNA and replaced by either wildtype GFP-CtIP or GFP-CtIPT847A in U2OS cells. HeLa were found to be sensitive to increased levels of CtIP, therefore we used U2OS cells for experiments involving ectopic expression of CtIP. U2OS cells were shown to react similarly to CtIP depletion compared to HeLa cells (Figure S4). S-phase cells were irradiated to form ICLs and stained for RPA2. GFP-CtIPT847A accumulated at ICL containing laser tracks comparable to wildtype levels, however RPA2 accumulation was impaired relative to cells expressing wildtype GFP-CtIP (Figure 6C). The percent cells containing bright RPA2 tracks was reduced 7.9 fold in cells expressing the CtIP phosphorylation mutant (8%) relative to control cells (63%) (n = 20 and 20 cells respectively, P value = 0.03) (Figure 6D).

We next examined whether reduced RPA accumulation at ICLs in CtIP depleted cells was associated with reduced ATR recruitment. The percent cells containing bright ATR signal along ICL containing laser tracks was reduced over 7 fold in CtIP depleted cells (8%) relative to control cells (58%) (n = 24 and 34 cells respectively; P value = 0.003) (Figure 6E, 6F). The requirement of CtIP for both RPA and ATR localization to ICL containing chromatin suggests that CtIP acts early in ICL repair prior to ssDNA generation and ICL incision.

### BRCA1 is required for CtIP accumulation at ICLs

**Figure 7:**
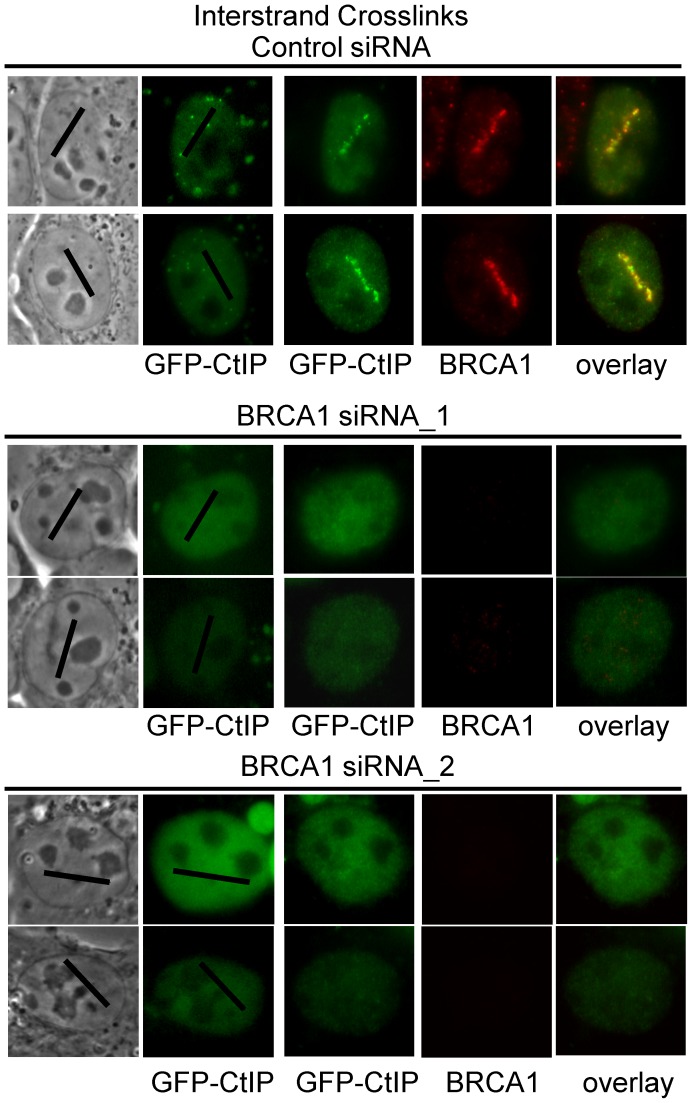
CtIP accumulation at ICLs is BRCA1–dependent. GFP-CtIP in S-phase control and BRCA1 depleted cells pre-and post-microirradiation with 730 nm light in the presence of 8-MOP.

BRCA1 ubiquitinates CtIP and is required for its localization to DSBs [79], [80]. In order to further characterize the requirements for CtIP recruitment to ICLs we examined whether BRCA1 was required for GFP-CtIP accumulation at ICLs. Cells were either BRCA1 depleted using two previously described independent siRNAs [81], [82] or treated with control siRNA. Endogenous CtIP was depleted by siRNA and CtIP expression reconstituted with silencing resistant GFP-CtIP. GFP-CtIP accumulation at ICL containing laser tracks was examined in S phase siRNA treated cells. CtIP accumulation at ICLs was found to be dependent on the presence of BRCA1 (Figure 7). In 3 independent experiments all cells (>25) treated with BRCA1 siRNA that lacked visible BRCA1 expression as verified by immunofluorescence staining and imaging, did not contain visible GFP-CtIP along the micro-irradiated region. In contrast, all control siRNA treated cells (>25) that had robust BRCA1 expression levels also contained GFP-CtIP at the ICL containing laser tracks. These results demonstrate that BRCA1 is required for CtIP recruitment to ICLs.

### CtIP is recruited to ICLs in a FANCM–dependent manner

**Figure 8:**
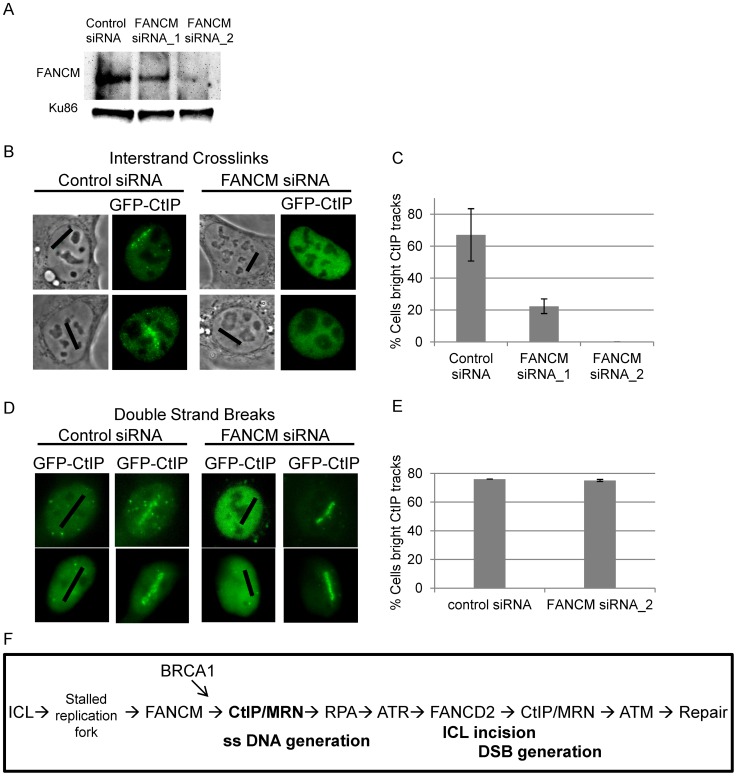
FANCM depletion reduces CtIP accumulation at ICLs but not at DSBs. (A) Immunoblot of control cells and cells depleted for FANCM. (B) GFP-CtIP in S-phase control and FANCM depleted cells pre-and post-microirradiation with 730 nm light in the presence of 8-MOP. (C) Quantification of cells scored as having visible GFP-CtIP along laser tracks in control or depleted cells. Bars indicate standard deviation between 3 independent experiments. (D) GFP-CtIP in S-phase control and FANCM depleted cells pre- and post-microirradiation with green laser to generate DSBs. (E) Quantification of cells scored as having bright GFP-CtIP along DSB containing laser tracks. (F) Model of CtIP function in replication associated ICL repair. FANCM binds ICL stalled replication fork and remodels fork to enable CtIP and MRN access. BRCA1 ubiquitinates CtIP facilitating its localization to damaged chromatin. CtIP and MRN act to generate ssDNA at a stalled fork. RPA bound single stranded DNA activates ATR. ATR phosphorylates FANCD2 followed by FA core complex ubiquitination of the FANCI-FANCD2 complex. The monoubiquitinated FANCI-FANCD2 complex localizes to the damaged chromatin and facilitates ICL incision and downstream repair events. CtIP and MRN act again to resect ends, activate ATM, facilitate completion of repair by homologous recombination.

FANCM has been shown to be required for RPA loading at ICLs as well as for activating the S-phase checkpoint [16], [83]. Data presented in Figure 6 indicates that CtIP is required for both RPA and ATR accumulation at laser activated ICLs in replicating cells. In order to determine when CtIP acts relative to FANCM we examined whether FANCM is required for CtIP localization to ICLs. U2OS cells stably expressing GFP-CtIP in which endogenous CtIP is depleted were treated with two independent FANCM siRNA s or control siRNA. Depletion was confirmed by immunoblot (Figure 8A). Cells were microirradiated to form ICLs and CtIP accumulation was examined. The percent of cells containing GFP-CtIP along ICL containing laser tracks was significantly reduced in FANCM depleted cells (22% FANCM siRNA_1, 0% FANCM siRNA_2) relative to control cells (67%) (n = 26, 11 and 30 cells respectively; P value = 0.0002) (Figure 8B, 8C). Next we examined whether FANCM was required for GFP-CtIP recruitment to DSBs. Cells treated with FANCM siRNA_2 or control siRNA were irradiated with 532 nm light to form DSBs and GFP-CtIP localization was monitored. In contrast to what was observed at ICLs, FANCM depletion did not have a significant effect on the percent of cells containing GFP-CtIP at DSB containing laser tracks (75% FANCM siRNA_2) relative to control cells (76%)(n = 20 and 17 cells respectively) (Figure 8D, 8E). This data suggests that FANCM acts upstream of CtIP and is required for its localization to ICL but not DSB containing chromatin.

## Discussion

The cellular response to ICLs involves the intersection of the DNA repair and replication checkpoint pathways. The FANCM/FAAP24 complex binds to, remodels and stabilizes stalled replication forks at an early step in ICL repair [13], [67]. FANCM/FAAP24 is also required for ATR mediated checkpoint activation in response to ICL stalled replication forks [15], [83]. In addition, FANCM deficient cells have decreased levels of FANCD2 monoubiquitination and chromatin bound FANCD2 [17], [84], [85]. We have shown that FANCM is required for CtIP localization to ICLs in replicating cells. CtIP in turn is required for proper accumulation of RPA, ATR and FANCD2 at ICLs. This places CtIP within the initiating steps of ICL repair, downstream of FANCM and upstream of RPA (Figure 8F). We propose the following model. First, FANCM/FAAP24 remodels ICL stalled replication forks to enable CtIP access. BRCA1 interacts with and ubiquitinates CtIP enabling it to bind to the damaged chromatin. Next, CtIP, presumably in concert with MRN, supports initiation of resection of the lagging strands of the previously active replication fork [59], [62], [63]. This resection activity is dependent on CDK phosphorylation site T847 on CtIP. ssDNA-RPA provides a platform for ATR-ATRIP binding. Once localized to the stalled fork activated ATR promotes S phase checkpoint activation and phosphorylates the FANCI-FANCD2 complex. The phosphorylated FANCI-FANCD2 complex is then monoubiquitinated, localized to damaged chromatin to facilitate ICL incision and generation of a DSB repair intermediate. This proposed order of events is consistent with that described for *Xenopus extracts* in which replication dependent ICL repair initiates at an ICL stalled fork, followed by generation of ssDNA, activation of ATR as measured by Chk1 phosphorylation, and excision of the crosslink in a FANCI-FANCD2 dependent manner [11], [18].

Cell cycle distribution and rate of BrdU incorporation are unaffected in CtIP depleted HeLa cells compared to control cells. This suggests that ICL processing defects observed in CtIP depleted cells are due to a deficiency in initiation of repair as opposed to an inhibition of cell cycle progression. The difference observed in cell cycle distribution of our CtIP depleted cells compared to those observed in mouse fibroblasts [70] are likely due to a difference in cell type as well as to a difference in the timing and method used to deplete CtIP.

A function for CtIP prior to ICL incision, and upstream of a repair intermediate that contains double stranded ends, is supported by the observation that CtIP depleted cells show a striking reduction in the accumulation of the DSB markers ATM-pS1981 and γH2AX at laser generated ICL tracks. In contrast, the accumulation of ATM-pS1981 and γH2AX at DSBs produced by direct laser irradiation are not affected by CtIP depletion. The effect of CtIP depletion on γH2AX at DSBs is in agreement with previously published observations in CtIP depleted U2OS cells [59]. This indicates that initiation of DSB repair, is not grossly affected by CtIP depletion. These results do not preclude an additional downstream role for CtIP where it may act together with MRN in a manner analogous to its function at directly generated DSBs to facilitate the processing of the double stranded DNA ends produced following ICL incision.

In summary, our data supports a model in which CtIP functions early in replication associated ICL repair downstream of FANCM and is required for the accumulation of RPA, ATR, FANCD2, ATM-pS1981, and γH2AX at ICLs. Thus, CtIP plays a critical role in initiating the DNA damage response at ICL stalled replication forks, prior to the generation of a double stranded DNA repair intermediate. It will be of interest to see how CtIP and MRN are regulated at ICL stalled forks and whether this response is different from that observed at other stalled forks in which helicase uncoupling contributes to checkpoint activation.

## Materials and Methods

### Laser microirradiation

Robolase III (RLIII) is a multi-modality laser ablation system that is based on a femtosecond pulsed Ti:Sapphire laser (Mai Tai, Spectraphysics, Newport Corp., Mountain view, CA) and a motorized inverted microscope (Axiovert 200 M, Zeiss) that utilizes a custom Labview-based software package developed specifically for the Robolase series of microscope systems [86]. In this study, the laser wavelength was set to 730 nm. The effective wavelength at the focal point is 365 nm at which psoralen is activated by a 2-photon mechanism [87]. The pulse duration was estimated at 200 fs and the repetition rate was 76 MHz. The laser was focused by a Zeiss 63×/1.4 plan-apochromat PH3 oil objective with measured transmission of 67% at 730 nm using the double-objective method to determine transmission [88]. The power used in this study was 2 mW before the objective which yielded a peak irradiance of 2.8×10^10^ W/cm^2^ at the focal point. For each cell, a 10 µm by 636 nm region of interest (ROI) was chosen inside the nucleus using real-time phase contrast imaging. An image of the designated ROI was recorded as reference. Laser exposure over each 10 µm long ROI was performed four times over each ROI with a total energy of 2.4 mJ. H2AX phosphorylation was used as a marker to determine the minimum power that provided a signal in the presence of 8-MOP. Double strand breaks were generated with the Robolase II (RLII) system that uses a frequency doubled 532 nm12 ps pulsed Nd:YVO_4_ laser [62], [86]. The ROI for each cell was 10 µm by 464 nm. The laser power used in this study was 5 mW in the focal spot for an irradiance of 2.3×10^9^ W/cm^2^ and the total energy deposited along the laser track inside each cell was 3.2 mJ. For experiments in which DSBs were generated cells were laser microirradiated on the same dish as cells treated to form ICLs and fixed at 2 hours 10 minutes post laser.

### Cell culture, siRNA transfection, and survival assays

Asynchronous or rapidly proliferating HeLa and HEK293 cells were maintained in Dulbecco's modified Eagle's medium (Invitrogen, Carlsbad, CA) supplemented with 10% bovine calf serum L-glutamine, and sodium pyruvate) at 37°C and 5% CO2.

Small interfering double stranded RNAs (siRNAs) were introduced into HeLa cells by transfecting cells in a 6 well dish with 50 pmol siRNA, and 5 µL Dharmafect 1 (Dharmacon, Lafayette, CO) per reaction. The following synthetic siRNAs used were obtained from Dharmacon, siLuciferase (control) and CtIP (siCtIP_1 and siCtIP_2) [59], FANCM_1 (GCAAAGUAGCCUAAAGAAAUU), FANCM_2 [83], siMUS81 (D-016043) and siRNA pool containing 4 siRNAs targeting XPF (siERCC4 D-019946-01.) The following siRNAs were obtained from Integrated DNA Technologies, BRCA1_1 (AATGCCAAAGTAGCTAATGTAUU) [82] and BRCA1_3 (AAGGAACCUGUCUCC ACA AAG UU) [81].

HEK293 cells were transfected with the indicated siRNA using Dharmafect 1, sixteen hours later the cells were washed with sterile PBS and fresh media was added. After an eight hour recovery the cells were trypsinized and seeded at 10^4^ per well in a six well plate. After 12 hours 10 µg/ml 8-MOP or angelicin were added to the cells. After 30 minutes incubation the cells were then exposed to the indicated amount of UVA radiation at approximately 360 nm using a Stratalinker (Stratagene, La Jolla, CA). Alternatively, cells were treated with indicated amount of MMC for 2 hours. Post treatment the cells were washed with sterile PBS, fresh media added and replaced every three days. On day 8 the cells were trypsinized and viable cells were counted using a CASY cell counter (Scharfe systems).

### GFP-CtIP expressing cells

A retroviral expression vector containing EGFP-CtIP (pBabepuro) was infected into U2OS cells for 48 hrs, followed by puromycin selection for 2–3 days to generate stable cell lines. For transient expression endogenous CtIP was first depleted by transfecting CtIP siRNA_1 into cells. Cells were transfected 24 hours later with silencing resistant GFP-CtIP using Effectene [59](Qiagen). Experiments were performed 24 hours post GFP-CtIP transfection. GFP-CtIPT847A was generated using Quikchange Site Directed Mutagenesis Kit (Agilent Technologies).

### Quantitative RT–PCR

Total RNA was isolated from control or CtIP siRNA transfected cells 48 hours post transfection using the RNeasy Kit (Qiagen). Target RNA was amplified using the Bio-rad iScript One Step RT-PCR kit with SYBR green kit (Bio-rad, Hercules, CA) on a Bio-rad real time quantitative PCR machine. The One step kit was used according to manufacturer's directions with the exception of 20 µL reactions, 2 ng total RNA per reaction, and 2 µL of each primer (5 uM stock). The following primers were used, housekeeping gene PBGD was amplified as a control (PBGDF- TCC AAG CGG AGC CAT GTC TG, PBGDR-  AGAATC TTG TCC CCT GTG GTG GA) CtIP (CTIPF- AAG AGG AGG AAT TGT CTA CTG C, CTIPR- AGA ATC TTG TCC CCT GTG GTG GA). Reactions were run on Chromo-4 qPCR I system (MJ Research, Waltham, MA). CtIP RNA depletion was verified to be between 60 and 70% by RT-QPCR for CtIP siRNA 1 and CtIP siRNA 2 in all experiments relative to control depleted cells.

### Bromodeoxyuridine incorporation assay

HeLa cells were transfected with siRNA as described above and seeded onto coverslips in 12-well plates. Thirty-six hours later, 2 mM thymidine was added to cultures for 16 hours. Cells were released from the thymidine block by washing 2 times with fresh media containing 100 uM bromodeoxyuridine (BrdU). Cells were incubated in BrdU containing media for 20 minutes to label S phase nuclei.. Cultures were fixed with 3.7% formaldehyde, stained with mouse monoclonal anti-BrdU antibody coupled to FITC (eBioscience, San Diego, CA), and counterstained with DAPI. ImageJ software was used to determine the total number of nuclei, BrdU-positive nuclei, and relative levels of fluorescence in individual cells.

### Cell cycle analysis

siRNA and control cells were prepared for flow cytometry analysis 48 hours post transfection. Cells were treated with trypsin/EDTA and washed twice in PBS before fixing them in 70% ethanol overnight at −20°C. Cells were then washed twice in PBS and incubated in staining solution (20 µg/ml propidium iodide, 20 µg/ml RNase A, 0.1% Triton X-100 in PBS) for 30 min at 37°C.Data were acquired on a BD FACS Calibur flow cytometer. Percentages of G_1_, S, and G_2_ phase cells were determined from cell cycle profiles by using the Watson pragmatic algorithm of the cell cycle platform within FlowJo software (Tree Star Inc., Ashland, OR) with the “remove doublets” and “remove debris” options enabled. *P* values were determined using an unpaired two-tailed *t* test.

### Immunofluorescence

Cells for laser microirradiation were cultured on gridded glass bottom dishes (Mattek, Ashland, MA) for 16 hours in 2 mM thymidine. Cells were washed twice with warm media to remove thymidine one hour prior to microirradiation and fresh media was added. Twenty minutes prior to laser microirradiation drugs were added to the cells; 10 µg/ml 8-MOP (MP Biomedicals, Solon, Ohio), or an equivalent molar amount of angelicin (8.6 µg/ml Sigma-Aldrich, St. Louis, MO).

Cells were allowed to recover for 2 hours post micoirradiation prior to fixation in all experiments except where noted. Soluble proteins were extracted in cytoskeletal buffer on ice for 5 minutes (10 mM Pipes pH6.8, 100 mM NaCl, 300 mM sucrose, 3 mM MgCl2, 1 mM EDTA and 0.5% TritonX-100) [89], followed by fixation in 3.7% formaldehyde phosphate buffered saline (PBS) at room temperature for 10 minutes, and permeabilized with 0.2% Triton-X. Cells were then stained with primary antibody in 3% BSA/PBS. The following primary antibodies were used in immunofluorescent staining: 1∶100 rabbit anti-RAD51 1∶100 (Abcam, Cambridge, MA), 1∶1000 mouse anti-γH2AX (Upstate Biotechnology, Waltham, MA), 1∶250 Rabbit anti-NBS1 NB100-143 (Novus Biologicals, Littleton, CO), 1∶200 Mouse anti ATM-pS1981 clone 10H11.E12 (Chemicon, Billerica, MA), 1∶200 Rabbit anti-FANCD2 NB100-182 (Novus Biologicals, Littleton, CO), 1∶50 Mouse Anti-RPA2 (Calbiochem, Gibbstown, NJ), 1∶100 Mouse anti-BrdU (ebioscience, San Diego, CA), 1∶500 Rabbit anti-RAD51 H92 sc-8349 (Santa Cruz Biotechnology, Santa Cruz, CA), 1∶1000 Mouse anti-MUS81 ab14387 (Abcam), 1∶500 XPF Ab17798 (Abcam), 1∶500 Rabbit anti-BRCA1 PA1-14072 (Thermo Fisher) 1∶1000 Rabbit anti-Ku86 SC9034 (Santa Cruz Biotechnology, Santa Cruz, CA). The following secondary antibodies were used at a 1∶5000 dilution in 3%BSA/PBS 1∶5000 Alexa Fluor 488 goat anti-mouse IgG (Molecular Probes, Eugene, OR) and 1∶5000 Alexa Fluor 594 goat anti-rabbit IgG (Molecular Probes, Eugene, OR). Nuclei were visualized by staining with 1 µg/ml Dapi (Invitrogen). Glass coverslips were mounted with Vectashield (Vectorlabs, Burlingham, CA). Samples were visualized and images acquired using a 63× objective on a Leica DM IRE2 microscope equipped with a Hamamatsu C4742-95 digital charge-coupled-device camera.

### Quantification of immunofluorescence

Images of cells were analyzed using Image J software (NIH, Bethesda, MD). The fluorescence along the microirradiated track was quantified in each individual cell as follows. Images were thresholded and converted into binary images. The same threshold was applied to all images from a single experiment. The number of pixels above threshold in 3 oval ROIs (150×50 pixels) were acquired per cell: one measurement for the area containing the laser track, and two measurements to determine the level of background signal in the nuclear region outside the laser track. The average number of background pixels was subtracted from the number of pixels measured along the microirradiated track for each individual cell. This resulted in a corrected pixel number for the intensity of a fluorescent signal along a laser track. For each positive control the average pixel number along the laser track was determined similarly. The average value from the positive control group per experiment was used to define the cut-off for experimental signals designated “bright” (corrected pixel number >50% of average positive control) or “low” (corrected pixel number <50% of average positive control). Each cell in the control and experimental siRNA group was then assigned a low signal or bright signal accordingly. The number of cells that fell into the “bright” and “low” categories were determined for each experiment. Cells which did not contain pan-nuclear γH2AX or RPA2 foci were considered to be outside of S-phase and were excluded from analysis. For analysis of GFP-CtIP expressing cells, cells were scored as positive if there was any detectable GFP-CtIP along laser track.

### Statistical analysis

Statistical analysis was performed using Microsoft Excel. Differences between control and experimental groups were considered statistically significant when the P-value, determined by a two-tailed unpaired Student's t-test, was ≤0.05.

### Immunoblotting

HeLa cells were lysed (10 mM Hepes pH 7.9, 10 mM KCl, 1.5 mM MgCl2, 0.34 M sucrose, 10% glycerol, 1 mM DTT, 0.1% triton, and protease inhibitors). Nuclear fraction was harvested by centrifugation at 1,399×g for 5 minutes. Pellet was resuspended in loading buffer, boiled for 5 mintues and put through 25 gauge syringe. Proteins were resolved by SDS-PAGE and transferred to nitrocellulose. Immunoblots were probed with indicated primary antibody, anti-FANCM (1∶1000) ab35620 (Abcam) and anti-Ku86 (1∶1000) (Santa Cruz). R. Baer provided a mouse monoclonal antibody to CtIP [90]. Membranes were washed, incubated with HRP-linked secondary antibodies (Invitrogen), and detected by chemiluminescence (Pierce, Fisher Scientific). For Mus81 and XPF blots cells were lysed by boiling in Laemmli buffer (4% SDS, 10% glycerol, 62.5 mM Tris pH6.8, 10% β-mercaptoethanol), and sonicated on ice. Immunoblots were probed with indicated primary antibody, anti-MUS81 (1∶1000) (Abcam) and anti-XPF (1∶1000) (Abcam), and anti-Ku86 (1∶1000) (Santa Cruz).

## Supporting Information

Figure S1 Effect of CtIP depletion on IR sensitivity. Survival of HEK293 cells transfected with control or CtIP siRNAs and exposed to IR. CtIP_1 and CtIP_2 are two independent siRNAs. Cells were irradiated 48 hours post transfection and survival was assessed 8 days post irradiation. Fraction surviving cells is calculated in respect to untreated cells.
(TIF)

Figure S2 Timecourse of γH2AX and RAD51 appearance at ICLs and DSBs. (A) S-phase, 8-MOP treated cells were fixed and stained for γH2AX and Rad51 at indicated times post microirradiation with 730 nm laser light. B, Cells were microirradiated with 532 nm laser light to generate double strand breaks. Cells were fixed and stained for γH2AX and Rad51 at indicated times post microirradiation. *Time point used in experiments.
(TIF)

Figure S3 MUS81 and XPF depletion do not affect H2AX phosphorylation at ICLs. (A) Left, γH2AX staining of S-phase control and MUS81 depleted cells 2 hours post microirradiation with 730 nm laser light in the presence of 8-MOP. Right, Quantification of γH2AX staining intensity along laser tracks in control (light grey) and Mus81 depleted cells (dark grey) microirradiated to form ICLs. Bar graph indicates percentage of total cells scored as having bright γH2AX signal along laser tracks. More than 30 cells were analyzed per condition. Bars indicate standard deviation between 3 independent experiments. (B) Left, γH2AX staining of S-phase control and XPF depleted cells 2 hours post microirradiation with 730 nm laser light in the presence of 8-MOP. Right, Quantification of γH2AX staining intensity along laser tracks in control (light grey) and XPF depleted cells (dark grey) microirradiated to form ICLs. Bar graph indicates percentage of total cells scored as having bright γH2AX signal along laser tracks. Bars indicate standard deviation. (C) Immunoblot confirmation of knockdown efficiency of MUS81 and XPF siRNAs. Ku86 is loading control.
(TIF)

Figure S4 CtIP depletion in U2OS cells reduces γH2AX at ICLs. (A) γH2AX staining of S-phase control and CtIP depleted cells 2 hours post microirradiation with 730 nm laser light in the presence of 8-MOP. (B) γH2AX staining of cell treated with CtIP siRNA and complemented with GFP-CtIP expression. (C) Quantification of γH2AX staining intensity along laser tracks in control, CtIP depleted, and CtIP depleted and GFP-CtIP complemented cells. Bar graph indicates percentage of total cells scored as having bright γH2AX signal along laser tracks (Over 15 cells were scored for each condition).
(TIF)
