# Supplementary figures and images for: CtIP Is Required to Initiate Replication-Dependent Interstrand Crosslink Repair
Source: PLoS Genet. 2012 Nov 8;8(11):e1003050. doi: 10.1371/journal.pgen.1003050 (PMC3493458; doi:10.1371/journal.pgen.1003050)

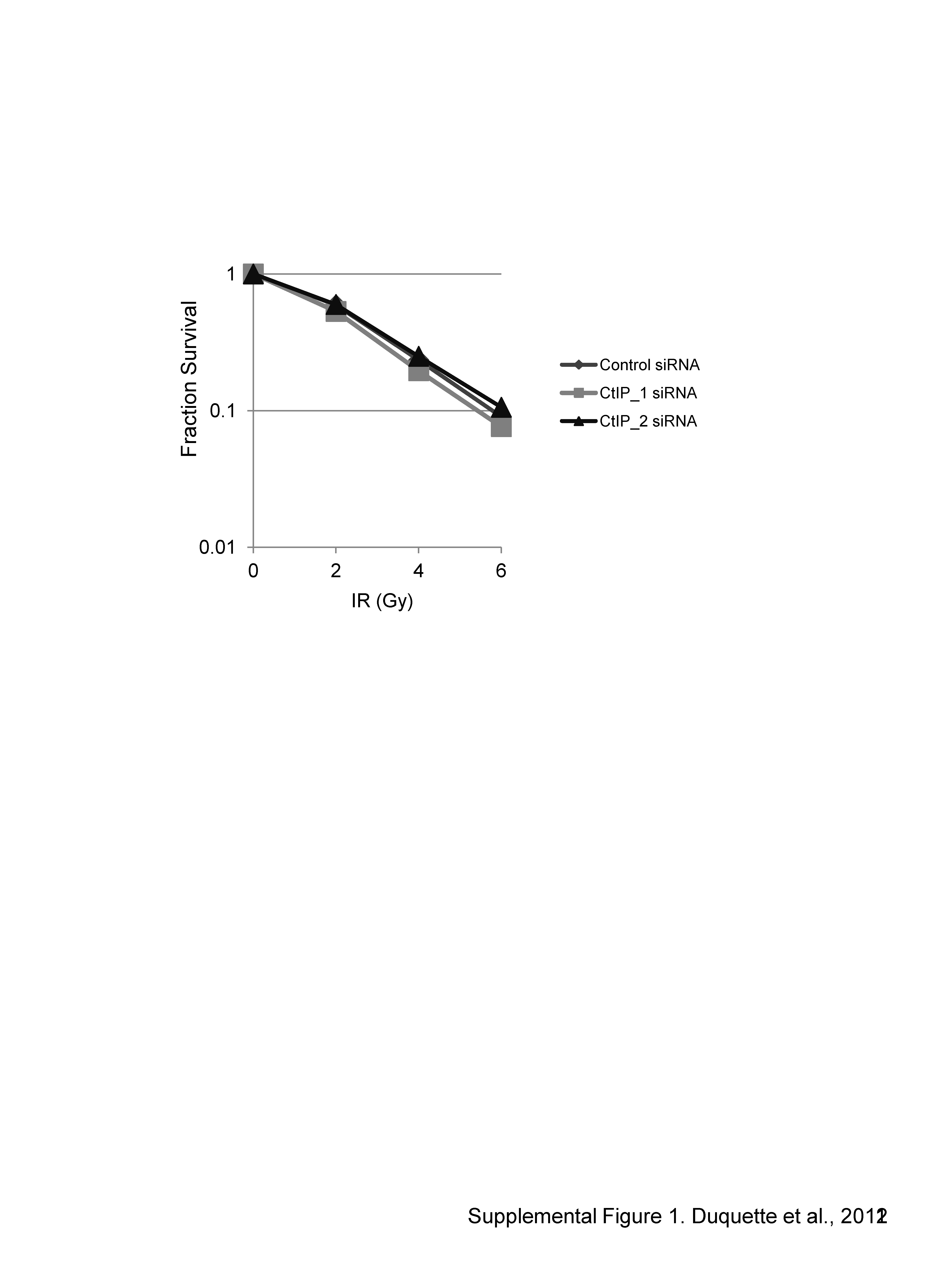

Supplement: Figure S1 — Effect of CtIP depletion on IR sensitivity. Survival of HEK293 cells transfected with control or CtIP siRNAs and exposed to IR. CtIP_1 and CtIP_2 are two independent siRNAs. Cells were irradiated 48 hours post transfection and survival was assessed 8 days post irradiation. Fraction surviving cells is calculated in respect to untreated cells. (TIF) [file pgen.1003050.s001.tif]

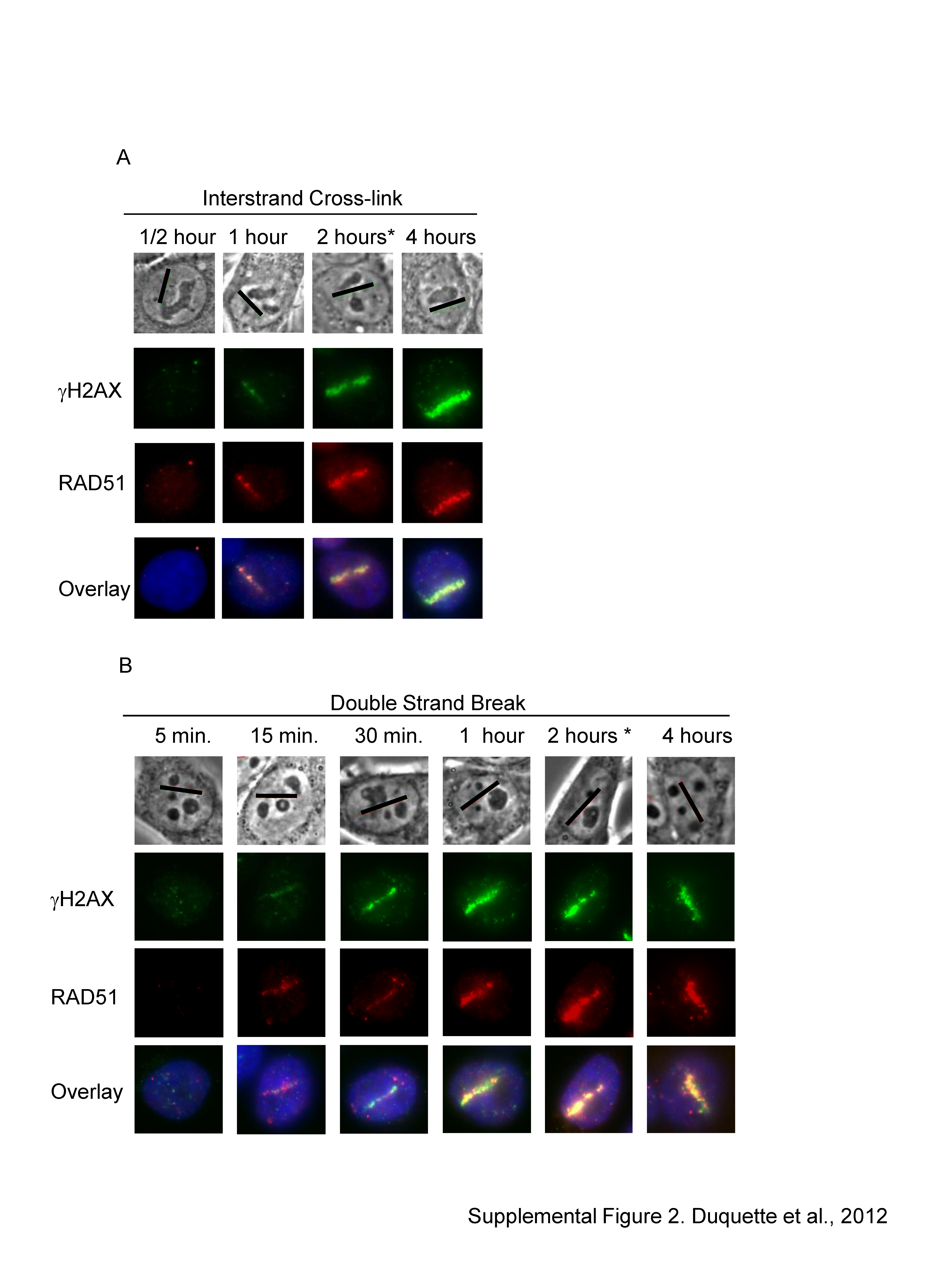

Supplement: Figure S2 — Timecourse of γH2AX and RAD51 appearance at ICLs and DSBs. (A) S-phase, 8-MOP treated cells were fixed and stained for γH2AX and Rad51 at indicated times post microirradiation with 730 nm laser light. B, Cells were microirradiated with 532 nm laser light to generate double strand breaks. Cells were fixed and stained for γH2AX and Rad51 at indicated times post microirradiation. *Time point used in experiments. (TIF) [file pgen.1003050.s002.tif]

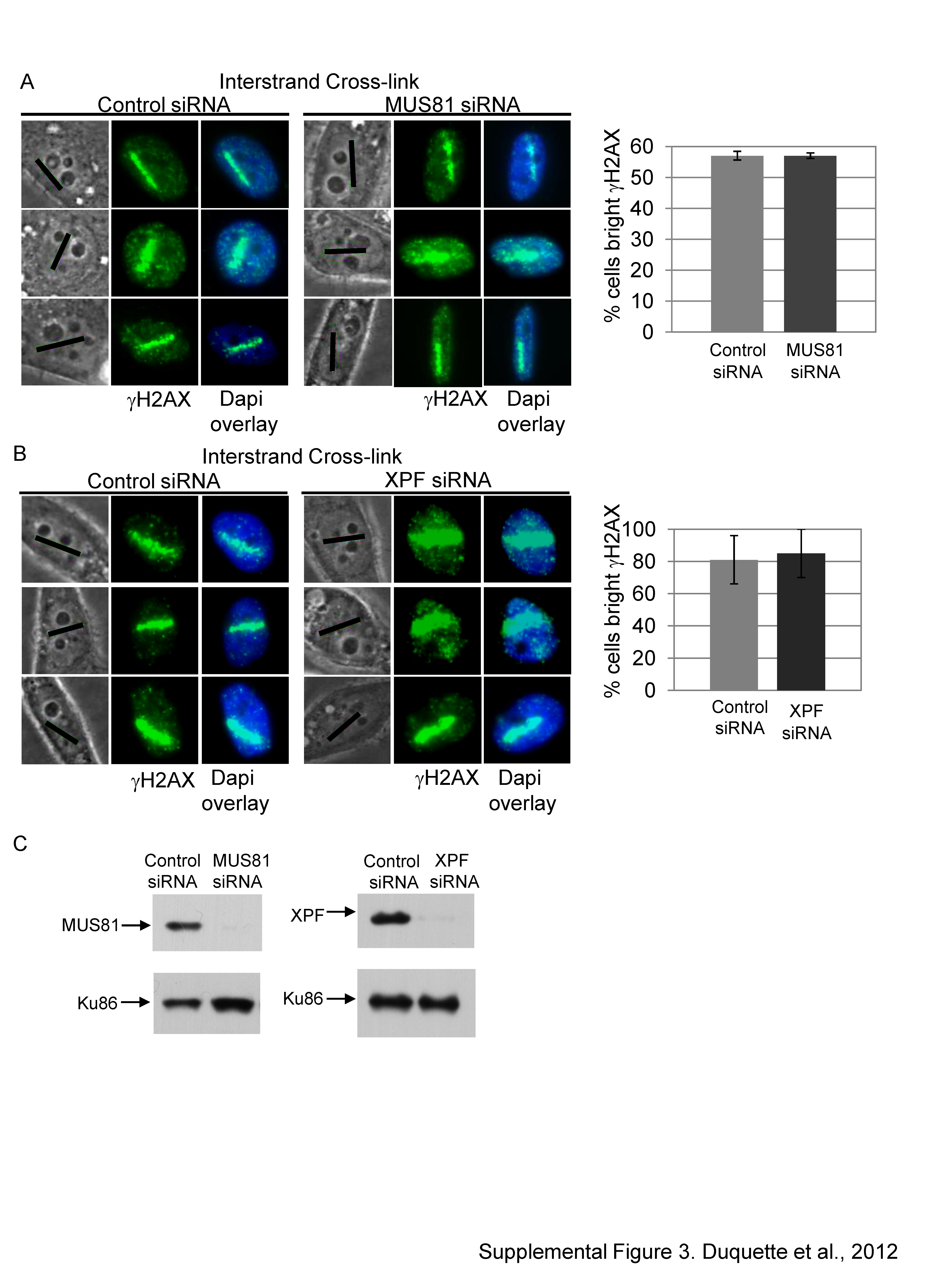

Supplement: Figure S3 — MUS81 and XPF depletion do not affect H2AX phosphorylation at ICLs. (A) Left, γH2AX staining of S-phase control and MUS81 depleted cells 2 hours post microirradiation with 730 nm laser light in the presence of 8-MOP. Right, Quantification of γH2AX staining intensity along laser tracks in control (light grey) and Mus81 depleted cells (dark grey) microirradiated to form ICLs. Bar graph indicates percentage of total cells scored as having bright γH2AX signal along laser tracks. More than 30 cells were analyzed per condition. Bars indicate standard deviation between 3 independent experiments. (B) Left, γH2AX staining of S-phase control and XPF depleted cells 2 hours post microirradiation with 730 nm laser light in the presence of 8-MOP. Right, Quantification of γH2AX staining intensity along laser tracks in control (light grey) and XPF depleted cells (dark grey) microirradiated to form ICLs. Bar graph indicates percentage of total cells scored as having bright γH2AX signal along laser tracks. Bars indicate standard deviation. (C) Immunoblot confirmation of knockdown efficiency of MUS81 and XPF siRNAs. Ku86 is loading control. (TIF) [file pgen.1003050.s003.tif]

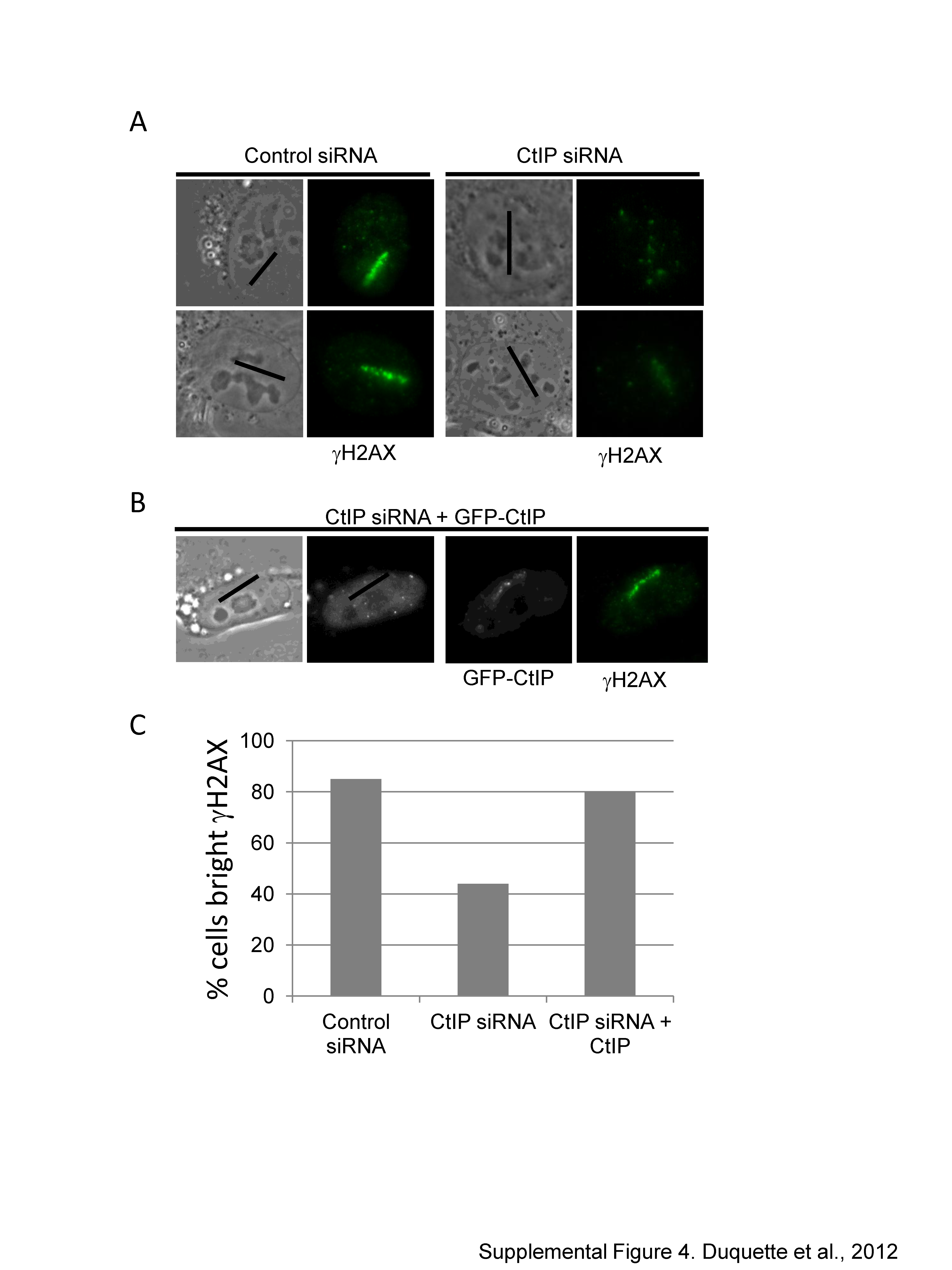

Supplement: Figure S4 — CtIP depletion in U2OS cells reduces γH2AX at ICLs. (A) γH2AX staining of S-phase control and CtIP depleted cells 2 hours post microirradiation with 730 nm laser light in the presence of 8-MOP. (B) γH2AX staining of cell treated with CtIP siRNA and complemented with GFP-CtIP expression. (C) Quantification of γH2AX staining intensity along laser tracks in control, CtIP depleted, and CtIP depleted and GFP-CtIP complemented cells. Bar graph indicates percentage of total cells scored as having bright γH2AX signal along laser tracks (Over 15 cells were scored for each condition). (TIF) [file pgen.1003050.s004.tif]
